# Novel Mathematical Model for Transient Pressure Analysis of Multifractured Horizontal Wells in Naturally Fractured Oil Reservoirs

**DOI:** 10.1021/acsomega.1c01464

**Published:** 2021-05-31

**Authors:** Yuan Gao, Md Motiur Rahman, Jing Lu

**Affiliations:** Petroleum Engineering Department, Khalifa University of Science and Technology, P.O. Box 2533, Abu Dhabi, United Arab Emirates

## Abstract

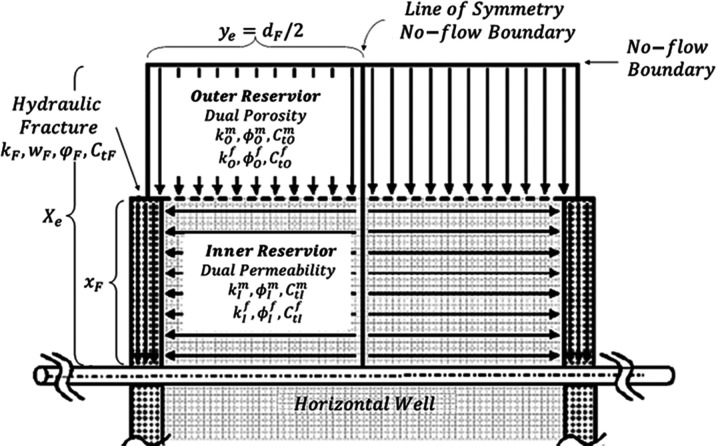

Multifractured horizontal wells have gained significant attention within the petroleum industry for commercial development. Despite considerable developments of transient pressure analysis or flow rate behaviors for horizontal wells in naturally fractured reservoirs, some significant problems are yet to be resolved, including high heterogeneity of reservoirs, pressure sensitivity of hydraulic fractures, and non-Darcy flow effect, which may occur during the production life. This paper presents a more pragmatic mathematical model for multifractured horizontal wells in naturally fractured reservoirs based on the fractal system, the theory of permeability modulus, and the time-fractional calculus correspondingly as an extension of the classic trilinear flow model. This new model comprises three modules: high heterogeneity of the reservoir based on the fractal system, the permeability modulus typically showing the pressure sensitivity of hydraulic fractures, and the anomalous diffusion describing non-Darcy flow turbulence. This investigation evaluates a trilinear dual-permeability dual-porosity flow model, with the dual-porosity model for the unstimulated outer reservoir flow region, the dual-permeability model for the stimulated inner reservoir flow region, and the permeability modulus for the flow region of hydraulic fractures. The comprehensive sensitivity analysis conducted indicates how the key parameters, such as fractal dimension, hydraulic fracture permeability modulus and conductivity, interporosity flow coefficient, storativity ratio, etc., affect the transient pressure behaviors, along with their reasons for the change in behavior. Application to a field case study further demonstrates the validity of the mathematical model, and the results presented may play a guiding role in well test interpretation.

## 1 Introduction

Increasing demand for oil and gas resources while being economical has significantly accelerated the development of multifractured horizontal wells (MFHWs). This technique has considerably enlarged the reservoir exposure and efficiently connected comparatively low-permeability regions in reservoirs with well-developed natural fractures. Hydraulic fractures are generally created to connect with existing natural fractures for more considerable productivity. Due to the complex hydraulic fractures communicated to natural fractures, flow behaviors through the porous reservoir and from the reservoir to the horizontal wellbore have become much more complicated.^1−4^

Previous researchers evolved their work from case studies to the field data analysis of transient pressure behaviors of MFHWs.^5−8^ Previous investigations have also incorporated computational methods to show the main flow regimes in the closed reservoir where the transversely fractured horizontal well completed, including the fracture linear flow, the bilinear flow, the early radial flow, the pseudo-radial flow, and the formation linear flow regimes respectively.^9,10^ It is evident that linear flow patterns are dominant in the whole production life of the reservoir, which is also the basic premise of the trilinear flow model.^11^

**Figure 1:**
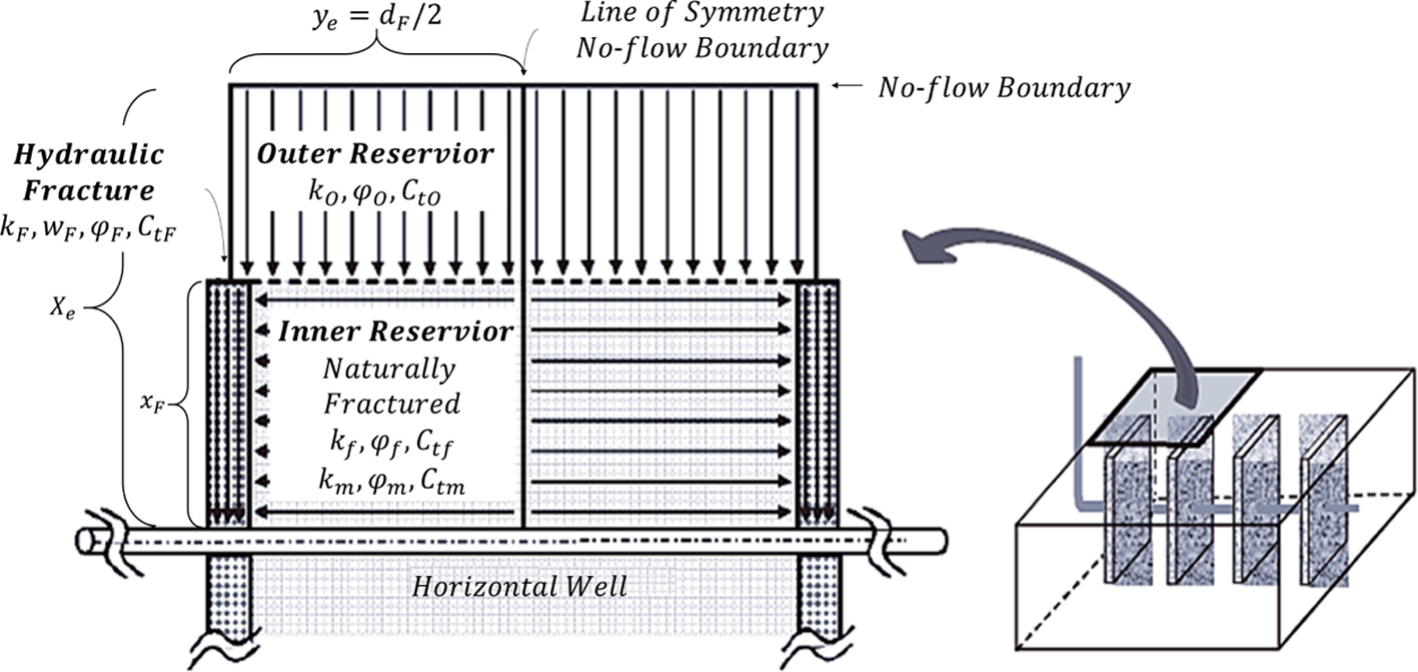
Trilinear dual-porosity flow model for the MFHW.

To cope with such a situation, Brown et al.^12^ first put forward a practical trilinear dual-porosity flow model for MFHW and took into account the effect of the outer reservoir flow region and the influence of natural fractures on fluid flowing behaviors using the dual-porosity model.^13^Figure 1 presents the outer reservoir flow region, regarded as an unstimulated homogeneous area with one single porosity and permeability; the stimulated inner reservoir flow region incorporates pseudo/transient interporosity flow from the matrix system to the fracture system; the region of hydraulic fractures comprises multiple equidistant transverse single-permeability/porosity hydraulic fractures with finite conductivity.

Although the model of Warren and Root^13^ is simple, it has proved versatile and has been widely used on account of the fact that the model incorporates all of the intrinsic properties of the matrix system, natural fractures, and hydraulic fractures and also reflects most of the flow regimes for MFHWs.^14^ The trilinear flow model indicates that the entire reservoir is divided into three flow regions where the linear flow is hypothesized and assumes that each flow region is homogeneous (except for the inner region of the reservoir where the dual-porosity model is introduced). However, this model, to some extent, could be undesirable for complicated cases of high heterogeneity, non-Darcy turbulence, and pressure sensitivity, which are common in naturally fractured reservoirs. Based on Brown’s model, a modified linear model was established for more complex reservoirs,^15^ where the reservoir is divided into five regions instead of three. For the Stalgorova and Mattar^15^ model, the outer reservoir flow region consists of two separate flow regions with different physical properties where the fluid flow shares the same flowing direction. The inner reservoir flow region replaces the dual-porosity model, and instead, it is subdivided into two homogeneous flow regions with different physical properties. Moreover, characteristics of the region of hydraulic fractures remain unchanged.

Jiang et al.^16^ presented a semianalytical model for pressure transient analysis for a hydraulically fractured horizontal well in a naturally fractured reservoir, considering non-Darcy and stress-sensitivity permeability effects. Here, the permeability modulus with stress-sensitivity effects is incorporated into mathematical models, resulting in nonlinearity of the equations. This nonlinearity is solved by application of the semianalytical method and by discretizing each hydraulic fracture into small segments. Then, the pressure response and pressure derivative type curves are generated to investigate the effects of non-Darcy and stress sensitivity.

Jiang et al.^17^ developed pressure response and pressure derivative type curves for improving the accuracy of horizontal well test interpretation, when the well is located in a naturally fractured-vuggy tight reservoir (but without a hydraulic fracture). They applied the finite element method to solve the bottom-hole pressure, using some internal boundary conditions. This nonlinear model includes the fractal index (fractal dimension and anomalous diffusion coefficient), showing nine different flow stages of dimensionless pressure and pressure derivative curves along the time group.

Zhao and Du^18^ also developed pressure response and pressure derivative type curves for a horizontal well in dual-porosity tight gas reservoirs without any hydraulic fracture. They solved the nonlinear partial differential equation and presented a mathematical model for unsteady flow between the matrix and natural fracture in inner and outer regions. Nine different flow regimes are defined based on pressure transient analysis type curves, which can be utilized for horizontal well test interpretations. Interestingly, they defined transfer regimes in inner and outer regions, where the shapes of pressure derivative and production rate derivative curves are dependent on in situ stresses.

Duan et al.^19^ developed type curves of pressure transient analysis of a horizontal well in a heterogeneous carbonate reservoir where homogeneous, dual-porosity (fracture-matrix), and triple-porosity (fracture-matrix-vug) systems are available. The interporosity flow on the pressure derivative curve is clearly identified including the linear flow, the pseudo-radial flow, and the pseudosteady-state flow in sequence. However, this linear composite model does not include the transverse hydraulic fractures in the horizontal well traversing a three-section reservoir.

An automated machine-learning smart model has been developed by Moosavi et al.^20^ who used a large number of pressure derivative curves (even with noisy data) from six distinct reservoir models to train and verify an artificial neural network. This trained neural network can analyze noisy pressure transient data from a horizontal oil well and convert pressure derivative data/graph and predict the formation properties of high-level accuracy. This neural network also did not include the transverse hydraulic fractures along the horizontal well.

The models mentioned above are pretty much accessible for analyzing transient pressure behaviors at a constant flow rate or investigating the transient flow rate at a constant bottom-hole flowing pressure. To some extent, these models, however, still have some limitations and would not be preferable for more complex cases, including the highly heterogeneous reservoir with well-developed natural fractures, the effect of non-Darcy flow turbulence, and pressure-sensitive transverse hydraulic fractures in a horizontal well traversing dual-porosity and dual-permeability tight oil formation.

Accordingly, this paper aims to present a more pragmatic mathematical model for MFHWs based on the classic trilinear flow model presented by Brown et al.^12^ This will also demonstrate how to resolve the problems in detail and will assist in the newly developed trilinear dual-permeability and dual-porosity (TDPDP) flow model.

Specifically, the primary objectives are listed as follows.

- The newly developed TDPDP flow model will integrate the dual-porosity model for the outer reservoir flow region and the dual-permeability model for the inner reservoir flow region and incorporate pressure sensitivity for the region of transverse hydraulic fractures.
- The new mathematical model will be derived in a complex scenario that considers high heterogeneity, pressure sensitivity of hydraulic fractures, and non-Darcy flow turbulence in the naturally fractured reservoir.
- The model analysis with key sensitive parameters and the flow regime division will be conducted on the basis of the newly developed TDPDP flow model. The study of a relevant field case will be performed for the rigorous TDPDP flow model, and the comparison of results between theoretical values and well test interpretation will be also provided.

### 1.1 High Heterogeneity Effect

Tight oil and gas reservoirs usually have a complex pore structure with randomly distributed natural fractures, which can be described by a fractal, statistical character. Fractal geometry is a method to characterize the statistical relation between a porous structure and length scale. This scaling exponent and fractal dimension can simplify the mathematical expression for depicting complex porous media with high heterogeneity. Thus, fractal dimension is an indicator that represents how much natural fractures occupy the space in irregular porous media and is a fundamental parameter to describe a fractal system (Cai et al.),^21^ which has been applied here to characterize the high heterogeneity briefly.

**Figure 2:**
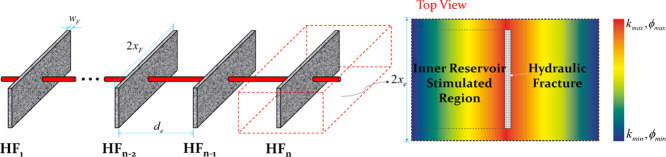
Schematic of permeability/porosity distribution in the stimulated region.

For naturally fractured reservoirs, high heterogeneity is a severe problem that cannot be easily neglected. During the fracturing process, the major hydraulic fracture gradually propagates in a predetermined direction so as to induce secondary fractures around it to communicate natural fractures initially developed in the target reservoir. As such, as illustrated in Figure 2, comparatively larger porosity or permeability may exist in the vicinity of the major hydraulic fracture and further distance away from the major hydraulic fracture could cause smaller porosity or permeability,^22^ which deviates the entire reservoir from homogeneity.

High heterogeneity is a predominant problem in naturally fractured reservoirs, and the fractal theory has been verified as a valid approach for the scenario of high heterogeneity.^23−25^ Since it was first successfully introduced in the analysis of transient pressure behavior,^26^ the fractal theory has been applied to the numerical model^27^ and the analytical model^28^ for the fractal-fracture network. This theory was then extended and combined with the trilinear flow model for the vertical well,^29^ which defined fractal porosity and permeability as follows (eqs 1 and 2).
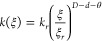
1
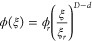
2Here, ϕ_*r*_ is the reference porosity (dimensionless). *k*_*r*_ is the reference permeability (mD). ξ_*r*_ is the reference distance (or regarded as the wellbore radius) (ft). ξ refers to the *x* or *y* axis. θ is the fractal connectivity index (dimensionless). *D* is the mass-fractal dimension (dimensionless). *d* is the Euclidean-embedding dimension (dimensionless). HF is the hydraulic fracture. The fracture half-length is *x*_F_, and the distance to the boundary from the well is *x*_e_.

This fractal system was successfully applied to the trilinear flow model for MFHWs.^22,30^ Wang et al.^22,30^ also presented a concept of stimulated reservoir volume (SRV) to describe flow characteristics of the dual-porosity medium and high heterogeneity encountered in the inner reservoir region, which has been verified on the basis of field data analysis and numerical simulation results. Accordingly, invariant porosity and permeability may cause a significant error for the naturally fractured reservoir in the end. It would be more appropriate that, rather than considered as a constant, the permeability and porosity in the inner reservoir flow region and outer reservoir flow region, to some degree, can be regarded as a function of a particular reference distance. This work will resolve the problem of the high heterogeneity effect using the fractal theory.

### 1.2 Non-Darcy Flow Turbulence

Darcy’s law is quite significant for the well test technique and primarily applied to reservoir numerical simulation. However, to some certain degree, Darcy’s law is only representative and sensible for some reservoirs with well-ordered fine grains.^2^ Since some disordered reservoirs, like reservoirs with highly well-developed natural fractures, may incorporate the anomalous diffusion process,^31,32^ the normal diffusion equation by Darcy’s law is no longer applicable for such a situation, which would always be encountered in the development of the highly disordered naturally fractured reservoir.

Conventional models of fluid flow through porous media typically depend on the validity of continuum methods. Limitations of the continuum assumption, however, become uncertain not only on account of high heterogeneity of the target reservoir, which is caused by varying scales of pores and throats and substantial dissimilarity between the matrix and fracture systems, but also due to the strong scale dependency and hereditary nature of the mean process variables in highly heterogeneous reservoirs. As such, reservoirs may display sharp discontinuity and random fluctuation in the velocity field, and Darcy’s law could not be feasible for such conditions.

An alternative is the model of anomalous diffusion for the non-Darcy flow through porous media.^33,34^ Regarding statistical concepts, diffusion is generally caused by the random Brownian motion of individual particles, and by and large, normal diffusion generally occurs in homogeneous porous rocks. Typically, this theory is more feasible for disordered structures, like highly heterogeneous reservoirs, which have been shown to be fractal in geometry.^26^ Fractal diffusion has been applied to take into account the stochastic nature of reservoirs’ heterogeneity, particularly those with highly disordered natural fractures. This method, then introduced into the trilinear flow model, brought in a time-fractional pressure derivative as expressed in the following equation rather than a non-Darcy coefficient. The time-fractional derivative can be computed as follows
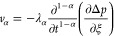
3Here, λ_α_ is a phenomenological coefficient, λ_α_ = *k*_α_/μ, 0 ≤ α ≤ 1; *k*_α_ is a phenomenological coefficient in anomalous diffusion, mD·h^1-α^/cP; and *v* is the velocity.

The theory of anomalous diffusion has been regarded as a practical alternative to conventional models for the non-Darcy flow effect in the classic trilinear flow model.^32,35^ After that, the theory of anomalous diffusion has been updated for nanoporous media^31,36^ and applied to more complex cases.^37^ This work adopts the theory of anomalous diffusion for non-Darcy flow in natural fractures.

### 1.3 Effect of Pressure-Dependent Permeability of Hydraulic Fractures

Hydraulic fractures tend to close due to the continuous decrease in hydraulic fracture pressure and increase in the effective stress on hydraulic fractures, resulting in a significant decline in the hydraulic fracture permeability. Accordingly, the permeability of hydraulic fractures must be rather considered as a function of pressure or pressure difference than a constant.^3,38,39^

A small negative influence would be exerted on the width of hydraulic fractures partly because of the proppant distributed along the hydraulic fractures, which sustains them from further deformation. Furthermore, as the pressure decreases in the hydraulic fracture, such a small relative change occurs in the width of hydraulic fractures, so it is realistic to regard the width as an invariant. It is generally recognized that the pressure-sensitive nature of hydraulic fractures is ubiquitous, showing that pressure drop in hydraulic fractures will result in fracture closure and permeability decrease.^40−42^ Thus, the assumption of constant permeability of hydraulic fractures must be replaced. Instead, permeability must be considered as a function of pressure or pressure difference rather than a constant. A much popular method to present the permeability change with respect to pressure or pressure drop is to introduce a permeability modulus of hydraulic fracture expressed as follows.^43^
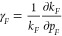
4Here, *γ*_F_ is the permeability modulus of hydraulic fractures, psi^–1^; *P*_F_ is the current pressure of hydraulic fractures, *psi*; and *k*_F_ is the permeability of hydraulic fracture at current pressure, mD. Performing integration on both sides, it yields
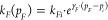
5Here, _Pi_ is the initial pressure, psi; and *k*_Fi_ is the permeability of hydraulic pressure at the initial pressure, mD.

The equation captures the feature of pressure sensitivity of hydraulic fractures, which has been widely used in the field.^44−46^ The permeability modulus, independent of pressure or pressure difference, reflects the effect of pressure sensitivity, ranging from 10^–4^ to 10^–3^ psi^–1^.^47^ Accordingly, this work focusses on forecasting the permeability of hydraulic fractures with the pressure change and analyze typical flow behaviors on the basis of the permeability modulus of hydraulic fractures.

## 2 Methodology

This section presents the mathematics for a trilinear dual-permeability dual-porosity (TDPDP) flow model in a complex scenario. This considers heterogeneity for each region (except for the region of the hydraulic fracture) by incorporating the fractal theory system, the non-Darcy flow turbulence by the principle of anomalous diffusion, as well as the pressure-dependent permeability of the hydraulic fracture by introducing a corresponding permeability modulus.

### 2.1 Physical Model and Basic Assumptions

**Figure 3:**
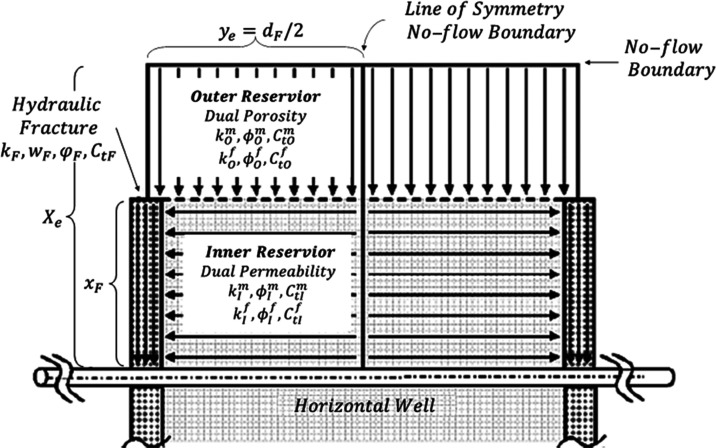
Schematic of the TDPDP flow model.

Based on the classic trilinear flow model by Brown et al.,^12^ this study, as illustrated in Figure 3, presents a modified trilinear flow model for the MFHW in the naturally fractured reservoir. This incorporates the dual-porosity model for the unstimulated outer reservoir flow region, the dual-permeability model for the stimulated inner reservoir flow region, as well as the pressure-sensitive hydraulic fracture region with finite conductivity. The dual-porosity model and the dual-permeability model share the same physical model but vary due to different flow patterns.

As shown in Figure 3, the TDPDP flow model presumes linear flow in three separate regions, which are coupled by the continuous flow rate and identical pressure system at the interface between contiguous regions. The necessary parameters and assumptions are listed as follows.

(1) Closed outer boundary (*x* = *x*_*e*_).
(2) Virtual impermeable interface (*y* = *y*_*e*_).
(3) Slightly compressible fluids and rock frame.
(4) Pseudo-interporosity flow between the matrix and fracture systems.
(5) Symmetric and equidistant identical transverse hydraulic fractures.
(6) Impermeable hydraulic fracture tips/end (*x* = *x*_*F*_).
(7) Half-length of the hydraulic fracture *x*_F_, constant.
(8) Width of the fracture *w*_F_, constant.
(9) Hydraulic fracture height *h*, constant.
(10) Permeability of the inner and outer reservoir regions, *k*_I_ and *k*_o,_ respectively.
(11) Porosity and total system compressibility,*ϕ* and *C*_*t*_, respectively.

For convenience in the process of derivation of the mathematical model in detail, it is necessary to list some useful dimensionless definitions, including dimensionless pressure, time, distance, reservoir conductivity, hydraulic fracture, and conductivity (Lee et al.).^48^

- Dimensionless pressure
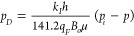
6
- Dimensionless time
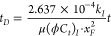
7
- Dimensionless distance
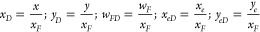
8;
- Dimensionless hydraulic fracture conductivity
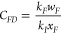
9
- Dimensionless reservoir conductivity
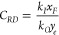
10

### 2.2 Mathematical Model

The mathematical model of the TDPDP flow model is derived in the Laplace domain concerning dimensionless parameters. The model derivation is developed for the outer reservoir flow region, the inner reservoir flow region, as well as the flow region of hydraulic fracture. What makes properties of both flow regions different is applying the dual-porosity model and the dual-permeability model for the outer and inner flow regions, respectively, since the fracturing technique stimulates the permeability of the matrix system in the inner reservoir flow region, the stimulated flow area, and has little effect on the outer reservoir flow region, the unstimulated flow area.

The fractal theory and the theory of anomalous diffusion are employed for both the outer and inner reservoir flow regions to characterize heterogeneity and non-Darcy turbulence effect in both flow regions, respectively. The pressure-sensitive or pressure-dependent permeability is merely considered in the region of hydraulic fracture on account of the larger fracture aperture in the hydraulic fracture by introducing a permeability modulus and establishing a corresponding power-law expression correspondingly. Fluid flow in the region of hydraulic fracture satisfies Darcy’s law. Table 1 summarizes permeability and porosity expressions based on the fractal theory and velocity expressions according to the theory of time-fractional anomalous diffusion for each flow region.

**Table 1:** Mathematical Expressions for Physical Properties of Each System and Region^a^

region		permeability	porosity	velocity
outer reservoir flow region	matrix			
	fracture			
inner reservoir flow region	matrix			
	fracture			
the region of HF				

a Note: superscripts *f* and *m* are the fracture system and matrix system, respectively. Subscripts: *D* is dimensionless form, *i* is initial condition, HF is flow region, *I* is inner reservoir flow region, *O* is outer reservoir flow region, *F* is hydraulic fracture flow region, and *r* is reference.

#### 2.2.1 Mathematical Model for the Outer Reservoir Flow Region

The dual-porosity model is integrated into the outer reservoir flow region, which sits beyond the tip of the hydraulic fracture, and the fluid flows in the *x*-direction, only satisfying the theory of anomalous diffusion. By the fractal theory, both the matrix system and the fracture system in this region are considered highly heterogeneous between which the pseudo-interporosity flow is assumed.

##### 2.2.1.1 Mathematical Model for the Matrix System

Fluid flow follows the theory of anomalous diffusion and the equation of mass conservation is expressed as eq 11. Each parameter is presented in the field unit, and the conversion coefficient is provided as well.
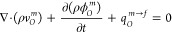
11where
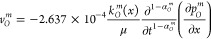
12
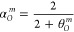
13Generally, the pseudo-interporosity flow from the matrix system to the fracture system is primarily recognized as a function of pressure difference between two systems. We introduce the theory of anomalous diffusion; the modified pseudo-interporosity flow rate *q*_*o*_^*m*→*f*^ from the matrix system to the fracture system in the outer reservoir flow region is typically concerned with the time-fractional derivative of the pressure difference between these two systems. For details, please refer to Appendix A.

The interporosity flow coefficient in the outer reservoir flow region λ_*O*_ is defined as eq 14.
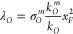
14The storativity ratio in the outer reservoir flow region ω_*o*_ is defined as eq 15.
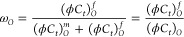
15The diffusivity coefficient η_*I*_ is expressed as eq 16 in the field unit.
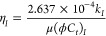
16The Laplace transform, first introduced for problems of the flow through porous media by van Everdingen and Hurst,^49^ is widely used for partial differential equations (PDEs). It aims to transform PDEs to ordinary differential equations (ODEs) in the broad sense, replacing the physical time, ***t***, by the Laplace time, or Laplace variable, ***s***. The transformed equation does make sense in the Laplace domain in the form of an ODE.

##### 2.2.1.2 Mathematical Model for the Fracture System

The mathematical model of the fracture system in the outer reservoir flow region also starts from the original equation of continuity expressed as eq 17. What deserves mention is that there is a negative sign instead of a positive one before the term of the modified interporosity flow on account of the fact that within the same period, the volume difference of fluid inlet and outlet must be identical to the fluid volume change subtracting the volume of interporostiy flow from the matrix system to the fracture system. For the fracture system, the flow term cannot be neglected anymore.
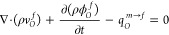
17where
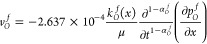
18
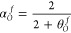
19Further substitutions and derivations with general solutions to the Bessel function and the outer and inner boundaries for the outer reservoir flow region are mathematically expressed in the dimensionless form in the Laplace domain. This has a long derivation comprising several sets of equations, not presented here. Thus, readers are advised to refer to Gao^50^ for detailed derivation of the equations.

**Outer Boundary Condition**
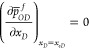
20**Inner Boundary Condition**
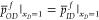
21

#### 2.2.2 Mathematical Model for the Inner Reservoir Flow Region

Integrating the dual-permeability model, the inner reservoir flow region sits between two adjacent hydraulic fractures. In this region, the fluid flows along the *y*-direction, perpendicular to the hydraulic fracture. What makes the dual-permeability model different from the dual-porosity model is that the flowing fluid is not only from the fracture system but also from the matrix system to the hydraulic fracture. The modified pseudo-interporosity flow is also assumed between these two flowing systems.

##### 2.2.2.1 Mathematical Model for the Matrix System

In the matrix system of the inner reservoir flow region, the flow term in the corresponding equation of continuity cannot be neglected anymore. The modified pseudo-interporosity flow is also assumed from the matrix system to the fracture system in the inner reservoir flow region. In the connection, the governing equation of the matrix system can be expressed as follows.
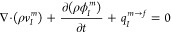
22where
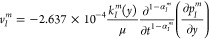
23
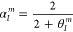
24The modified pseudo-interporosity flow rate *q*_*I*_^*m*→*f*^ from the matrix system to the fracture system in the inner reservoir flow region is defined, which is also related to the time-fractional derivative of the pressure difference between these two systems. For detailed derivations, please see Appendix B.

The outer and inner boundaries of the matrix system in the inner reservoir flow region are mathematically expressed in the dimensionless form in the Laplace domain as follows.

**Outer Boundary Condition**
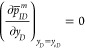
25**Inner Boundary Condition**
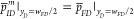
26

##### 2.2.2.2 Mathematical Model for the Fracture System

In this fracture system, the fluid flow follows the theory of anomalous diffusion and the corresponding equation of motion is expressed as eq 27. Each parameter is presented in the field unit and the conversion coefficient is provided as well.
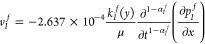
27where
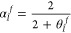
28It is assumed that the flowing fluid from the fracture system in the outer reservoir flow region would only flow into the fracture system in the inner reservoir flow region on account of less flow resistance between the two fracture systems resulting from the smaller permeability difference. In this region, a generalized 2D form of the governing equation is considered for the fracture system to incorporate the inflow term of the flowing fluid from the outer region to the inner region.

Following a similar derivation pattern with the governing equation of the fracture system in the inner reservoir flow region, the outer and inner boundaries of the fracture system in the inner reservoir flow region can be mathematically expressed in the dimensionless form as follows.

**Outer Boundary Condition**
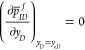
29**Inner Boundary Condition**
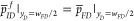
30

Please refer to Appendix C for the detailed governing equations of the fracture system in the inner region.

#### 2.2.3 Mathematical Model for the Hydraulic Fracture Flow Region

Flowing fluid in the hydraulic fracture follows Darcy’s law, and the flow occurs in the *x*-direction from the fracture tip (*x* = *x*_F_) to the horizontal wellbore (*x* = 0). The pressure-dependent permeability is taken into account in the region of hydraulic fracture. In the region, the governing equation is also given in a generalized 2D form to integrate the effect of the flowing fluid from the inner reservoir flow region to the region of the hydraulic fracture. In this connection, the governing equation can be expressed as eq 31.
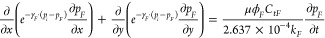
31Equation 31 can be rewritten in the dimensionless form as follows.
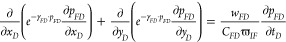
32where the dimensionless permeability modulus is defined as eqs 33 and 34.
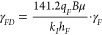
33
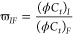
34Application of the linearization method and the perturbation theory and further derivation with the Tylor expansion are presented in Appendix D. At the interface between the inner reservoir flow region and the region of hydraulic fracture, the flow rate must be continuous and identical, and the mathematical formula can be written in the dimensionless form.^50^

The outer and inner boundary conditions for the region of hydraulic fracture can be mathematically expressed as follows.

**Outer Boundary Condition**
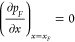
35**Inner Boundary Condition**
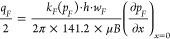
36In the Laplace domain, these two boundary conditions can be simplified in the dimensionless form, shown as follows.

**Outer Boundary Condition**
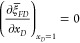
37**Inner Boundary Condition**
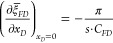
38With the boundary conditions, the final solution can be obtained as follows.
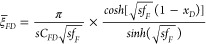
39The dimensionless bottom-hole pressure can also be calculated as *x*_*D*_ equals to 0, which is shown as follows.
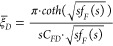
40

##### 2.2.3.1 Choking Skin Effect and Wellbore Storage Effect

**Figure 4:**
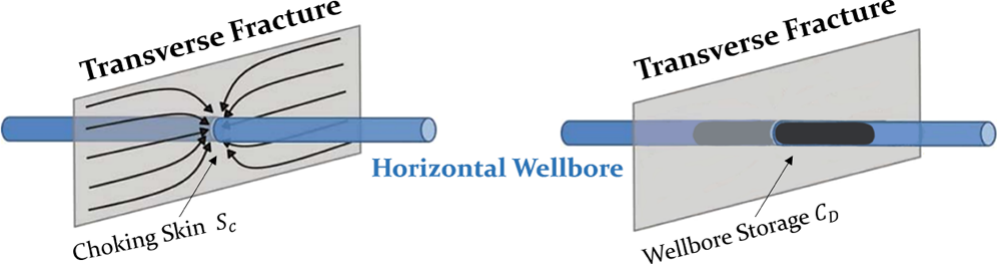
Choking skin effect and wellbore storage effect of the MFHW.

In the actual scenario of fluid flow into the horizontal wellbore illustrated in Figure 4, the choking skin effect and the wellbore storage effect area also taken into account.

As the choking skin factor is considered, the dimensionless bottom-hole flowing pressure can be rewritten as eq 41.
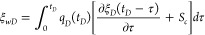
41where the choking skin factor can be expressed as
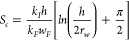
42The dimensionless bottom-hole flow rate *q*_*D*_(*t*_*D*_) in eq 41 can be expressed in terms of the dimensionless wellbore storage coefficient *C*_*D*_.
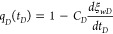
43By performing the Laplace transform and further derivations using the Stehfest numerical inversion expressed as eq 44, the final solution can be obtained in the actual physical domain.
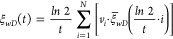
44where
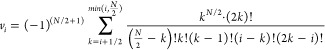
45For computational accuracy, *N* in eqs 44 and 45 must be selected from the sequence of even integers between 4 and 18. Finally, this leads to the expression of the dimensionless bottom-hole flowing pressure *p*_*wD*_ considering the pressure-dependent permeability of the hydraulic fracture in the actual physical domain.

##### 2.2.3.2 Numerical Differentiation of Dimensionless Bottom-Hole Flowing Pressure

In the well testing, the bottom-hole flowing pressure derivative with respect to time (*dp*_*wD*_/*dt*_*D*_) needs to be determined for the identification of flow regimes during the productive life of the target reservoir. The numerical differentiation is much more applicable due to the computation capacity of common computational tools. In this study, the following method of numerical differentiation will be adopted on account of the fact that not only does it ensure calculation precision but also it can substantially reduce the memory consumption of computers.^50^

The formula of numerical differentiation can be expressed as eq 46.
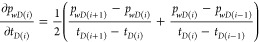
46where *i* = 2 ∼ (*n* – 1), and *n* here refers to the length of a given time *t*.

**For *i* = 1**
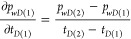
47**For *i* = *n***
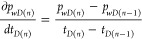
48

## 3 Model Validation

A field case from a multifractured horizontal well in a tight carbonate oil reservoir in the Bohai Bay region of Eastern China has been applied to validate the model. The target carbonate reservoir is abundant in low-viscous single-phase crude oil. The depth of the reservoir midpart is around 10 379.52 ft below the sea level, and the formation height is about 282.04 ft. The initial reservoir pressure is about 4273.12 psi, and the initial reservoir temperature is approximately 396.15 K. Outer boundaries of the target reservoir are entirely closed. The average reservoir permeability (prefracturing) is about 0.132 mD, and the average reservoir porosity stands at around 0.078.

The multifracture horizontal well is about 4125.65 ft in the total wellbore length and around 0.33 ft in the wellbore radius. Ten fracturing stages are equidistantly distributed along the horizontal wellbore, and hydraulic fractures penetrate the entire formation in the direction of formation height. Typical heterogeneity-related parameters, such as fractal dimension 2.05, Euclidean-embedding dimension 2.0, and fractal connectivity index 0.05, are considered. Crude oil is produced from the horizontal wellbore at a constant production rate. Other significant data are listed in Table 2.

**Table 2:** Basic Parameters for the Target Carbonate Reservoir in the Bohai Bay Region

parameters	values	parameters	values
reservoir boundary X (ft)	15 297.35	crude oil viscosity (cP)	9.58
permeability for the fracture system (mD)	2.59 × 10^3^	hydraulic fracture height (ft)	282.04
porosity for the fracture system (%)	0.83	initial average permeability of hydraulic fractures (mD)	27.11 × 10^3^
total compressibility for the fracture system (psi^–1^)	2.24 × 10^–3^	average porosity of hydraulic fractures (%)	0.46
permeability for the matrix system (mD)	7.85 × 10^–6^	total compressibility of hydraulic fractures (psi^–1^)	8.34 × 10^–3^
porosity for the matrix system (%)	3.62	average daily production rate (STB/D)	308.45
total compressibility for the matrix system (psi^–1^)	6.45 × 10^–4^	formation oil volume factor (bbl/STB)	1.13

### 3.1 Curve Matching for the Permeability of Hydraulic Fractures

**Figure 5:**
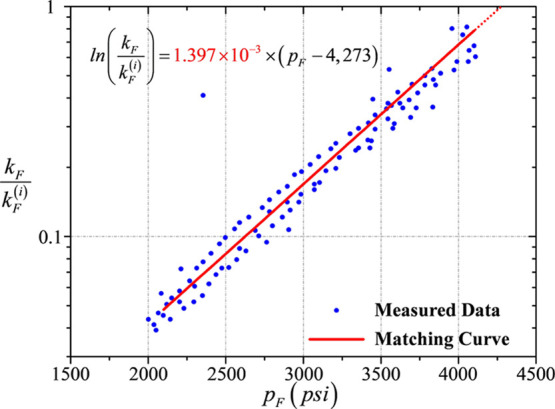
Data matching for the permeability modulus of hydraulic fractures.

The average permeability of hydraulic fractures is highly pressure-sensitive. The initial average permeability of hydraulic fractures (at the initial reservoir pressure) is around 27.11 × 10^3^ mD, and the declining trend of permeability with respect to the pressure drop in hydraulic fractures satisfies the exponential law. The permeability modulus of hydraulic fractures can be obtained by the ordinary least-squares technique to match the permeability data of hydraulic fractures from well logging with a straight line in a semilog coordinate system as illustrated in Figure 5. The figure shows that the slope of the red matching straight line typically refers to the average permeability modulus of hydraulic fractures, 1.397 × 10^–3^ psi^–1^ specifically.

### 3.2 Curve Matching for the Transient Pressure Data

**Figure 6:**
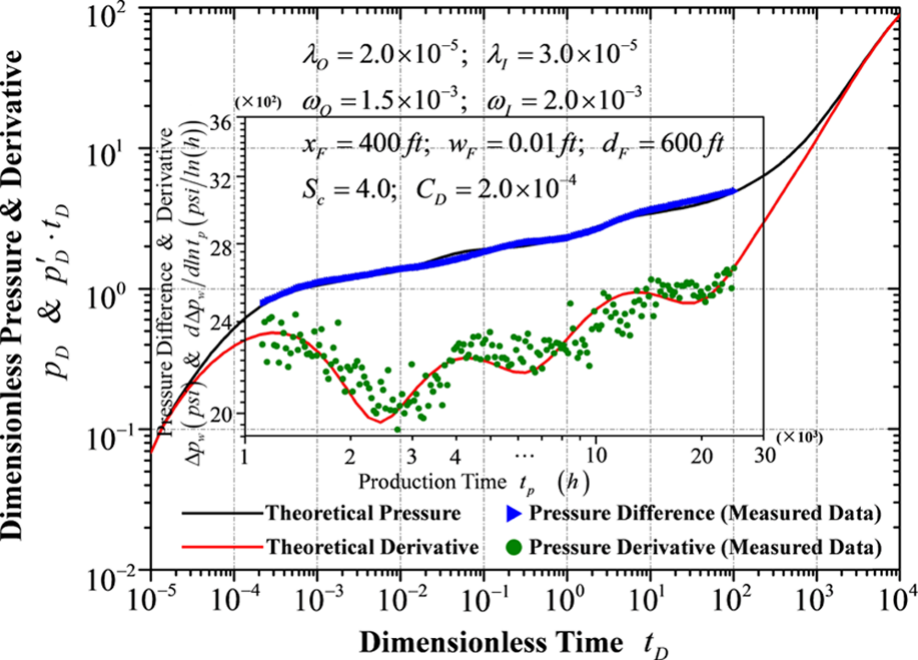
Data matching for transient pressure behaviors.

The data at the early flowing stage are removed on account of the flowing turbulence at this stage (the constant production rate is a basic prerequisite). The theoretical well test curves (solid lines) and measured transient pressure data (scatter points) are illustrated in Figure 6.

Meanwhile, the comparison between theoretical parameters and well test interpretation is listed in Table 3. Transient pressure data are converted into the corresponding dimensionless form by the dimensionless definitions. The “theoretical parameters” column lists the values for plotting the theoretical well test curves (solid black and red curves), while the “well test interpretation” shows the results of the well test interpretation. As can be seen, the relative errors between the corresponding values in these two columns sit within the engineering tolerance. In this connection, the model is also validated as a correct field-applicable one.

**Table 3:** Results Comparison between Theoretical Parameters and Well Test Interpretation

elements	theoretical parameters	well test interpretation
interporosity flow coefficient, λ	λ_O_ = 2.0 × 10^–5^	2.79 × 10^–5^
	λ_I_ = 3.0 × 10^–5^	
storativity ratio, ω	ω_O_ = 1.5 × 10^–3^	1.93 × 10^–3^
	ω_I_ = 2.0 × 10^–3^	
HF half-length, *x*_*F*_ (ft)	400	412.83
HF width, *w*_*F*_ (ft)	1.0 × 10^–2^	1.18 × 10^–2^
HF spacing, *d*_*F*_ (ft)	600	589.37
skin factor, *S*_*c*_	4.0	3.85
wellbore storage, *C*_*D*_	2.0 × 10^–4^	2.17 × 10^–4^

## 4 Results and Discussion

Model analysis, based on the dimensionless theoretical parameters, aims to observe how the well test curves are influenced by the hydraulic permeability modulus, the fractal dimension, the fractal connectivity index, the interporosity flow coefficient, the storativity ratio, the hydraulic fracture conductivity, the choking skin factor, and the wellbore storage coefficient separately. This is all about transient pressure analysis, where the reservoir is infinitely acting during the well tests.

### 4.1 Response to Different HF Permeability Moduli γ_FD_

**Figure 7:**
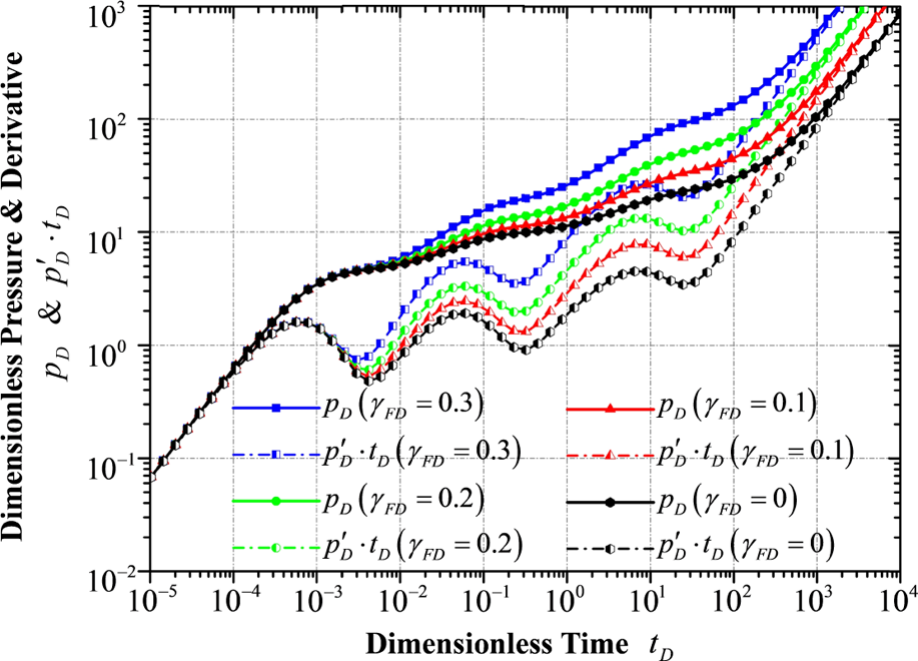
Transient pressure/pressure derivative response to different hydraulic fracture permeability moduli.

As the pressure of the hydraulic fracture decreases, the permeability declined in an exponential manner. As illustrated in Figure 7, the hydraulic fracture permeability modulus mainly exerts a significant effect on the whole production stages except for the stage of wellbore storage effect. The reservoir energy (pressure system) will be consumed faster as the permeability of the hydraulic fracture is more sensitive to pressure drop, leading to a more “going-up” trend in both the dimensionless pressure curve and the dimensionless pressure derivative curve.

### 4.2 Response to Different Fractal Dimensions *D*

**Figure 8:**
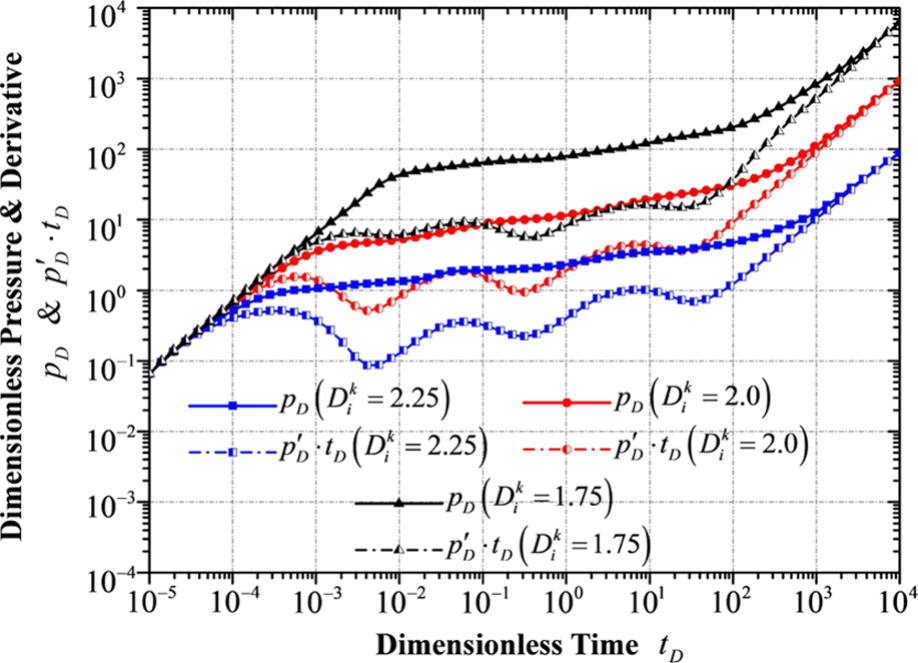
Transient pressure/pressure derivative response to different fractal dimensions of the fractal-fracture network.

Figure 8 shows the effect of the fractal dimension of the natural fracture network on the transient pressure behavior. Both the dimensionless pressure curve and the dimensionless pressure derivative curve are quite sensitive to the fractal dimension *D* because it is a measure of the complexity of the fracture network. A smaller fractal dimension contributes to a larger pressure response as it indicates the more complex geometric property of the natural-/induced-fracture networks and the much stronger heterogeneity of the entire reservoir. This requires a larger pressure difference to enable the fluid flowing through more highly disordered porous media. Also, a more highly disordered reservoir (a smaller fractal dimension) typically makes the stage of wellbore storage effect more dominant.

### 4.3 Response to Different Fractal Connectivity Indexes θ

**Figure 9:**
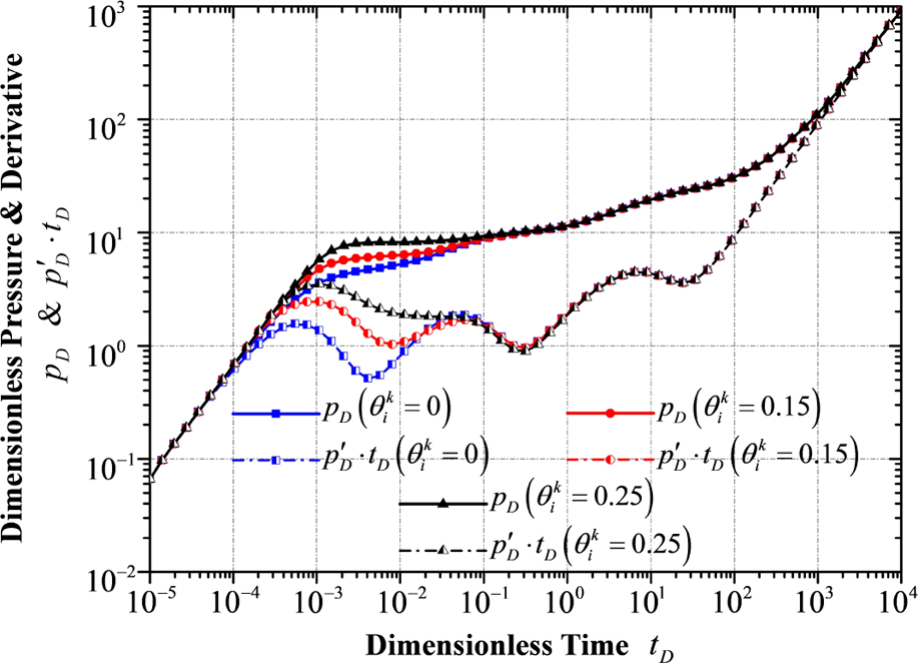
Transient pressure/pressure derivative response to different fractal connectivity indexes of the fractal-fracture network.

The exponent θ, the fractal connectivity index, generally characterizes the fractal diffusion of fluid through porous media from the outer reservoir flow region to the inner reservoir flow region and from the inner reservoir flow region to the flowing region of hydraulic fractures. In single-phase (specifically single-phase oil) diffusion processes, the dimensionless pressure and dimensionless pressure derivative at the early-time response (before the stage of the interporosity flow in the inner reservoir flow region) would increase as the fractal connectivity index θ increases (Figure 9). A larger fractal connectivity index indicates worse connectivity of the fractal-fracture network, showing a larger transient pressure response and a much more hindered diffusion process in the fracture network.

### 4.4 Response to Different Interporosity Flow Coefficients λ

**Figure 10:**
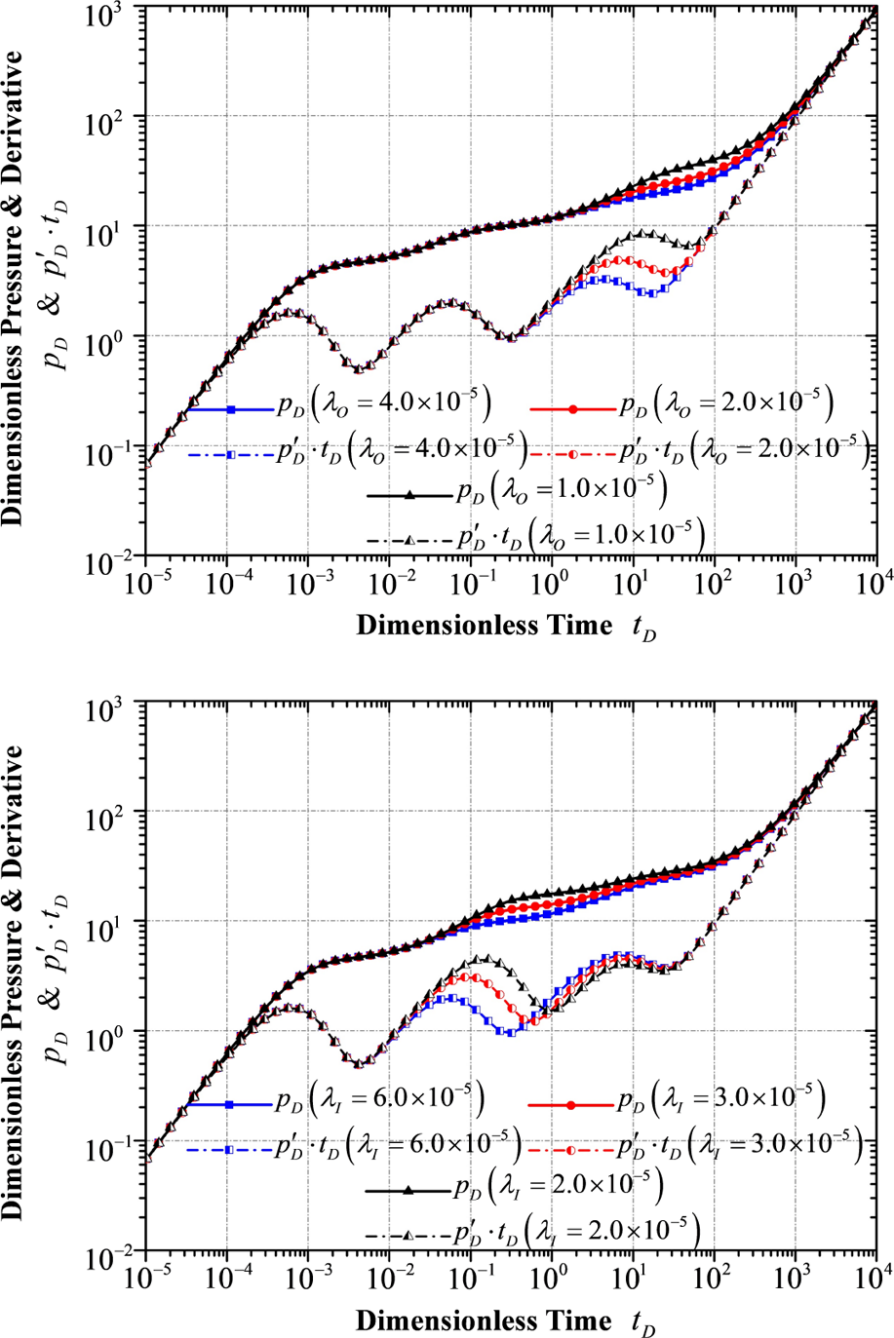
Transient pressure/pressure derivative response to different interporosity flow coefficients: (top) interporosity flow coefficient *λ*_O_ in the outer reservoir flow region, and (bottom) interporostiy flow coefficient *λ*_I_ in the inner reservoir flow region.

A smaller interporosity flow coefficient means a larger difference in permeability between the matrix system and the fracture system, which demonstrates a more significant resistance in the fluid exchange from the matrix system to the fracture system in both inner and outer reservoir flow regions. In this connection, as illustrated in Figure 10, the location of the hollow section moves toward the right-hand side with the interporosity flow coefficient decreasing. Therefore, a relatively larger pressure difference (Δp) is required to make the interporosity flow happen, and it will take a longer time to ensure that the pressure in the fracture system is identical to that in the matrix system, leading to a higher pressure difference and a higher pressure derivative with respect to time. It is evident that a smaller interporosity flow coefficient *λ*_*O*_ elongates the elapsed time of the flow period in the fracture system in the outer reservoir flow region. It is also easily observed that a smaller interporosity flow coefficient *λ*_*I*_ in the inner reservoir flow region causes an extension of the elapsed time for the flow in the fracture system of the inner reservoir flow region and contributes to flow period contraction in the outer reservoir flow region.

### 4.5 Response to Different Storativity Ratios

**Figure 11:**
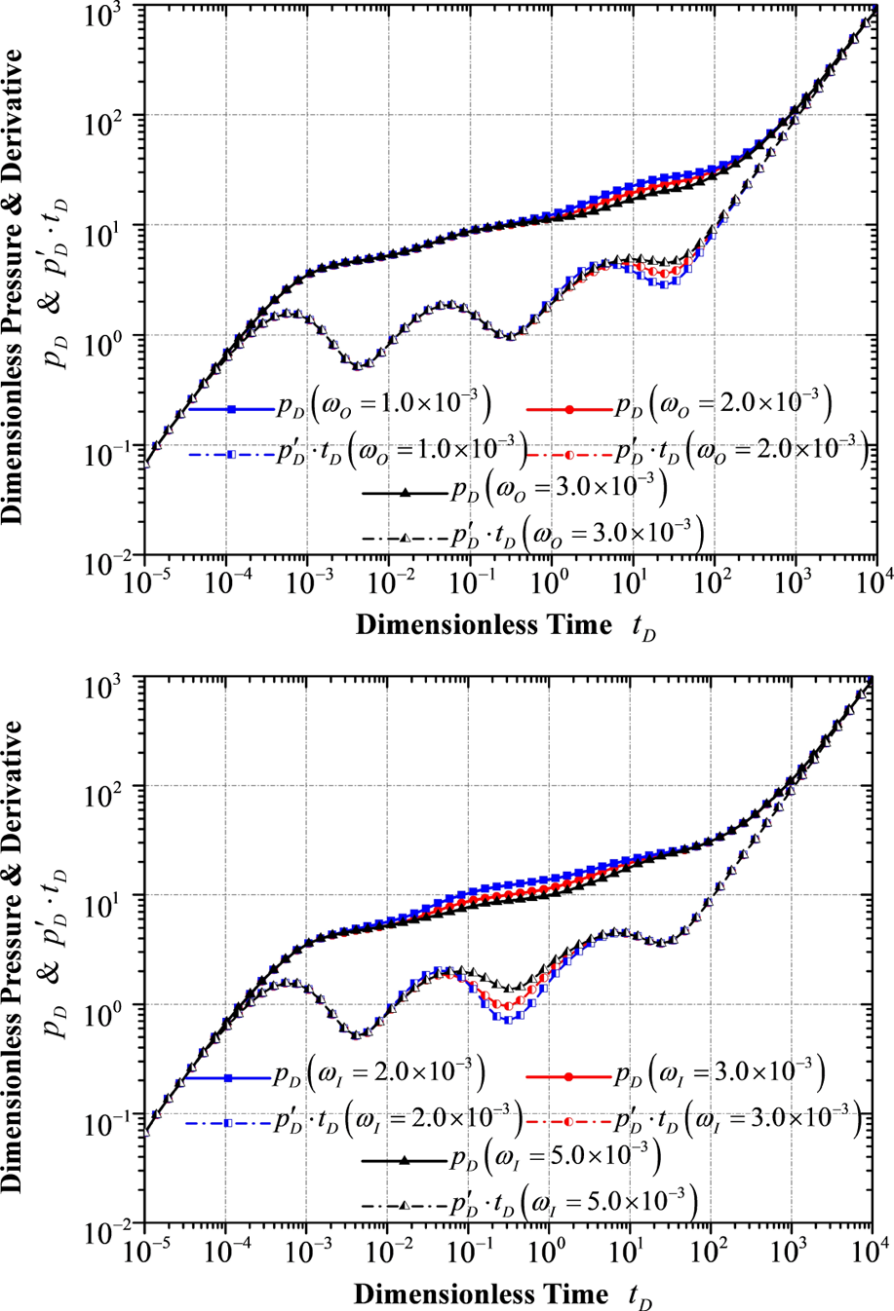
Transient pressure/pressure derivative response to different storativity ratios: (top) storativity ratio in the outer reservoir flow region, and (bottom) storativity ratio in the inner reservoir flow region.

As illustrated in Figure 11, the storativity ratio mainly influences the width and depth of the second and third “hollow” sections on the curve of the dimensionless pressure derivative, and the smaller the storativity ratio is, the wider and deeper the hollow section appears. That is on account of the fact that a smaller storativity ratio indicates less fluid in the fracture system where a more significant pressure drop happens in a shorter period of time. In this connection, a longer time is required to ensure a simultaneous pressure decrease in both matrix and fracture systems in both inner and outer reservoir flow regions.

### 4.6 Response to Different HF Conductivities *C*_FD_

**Figure 12:**
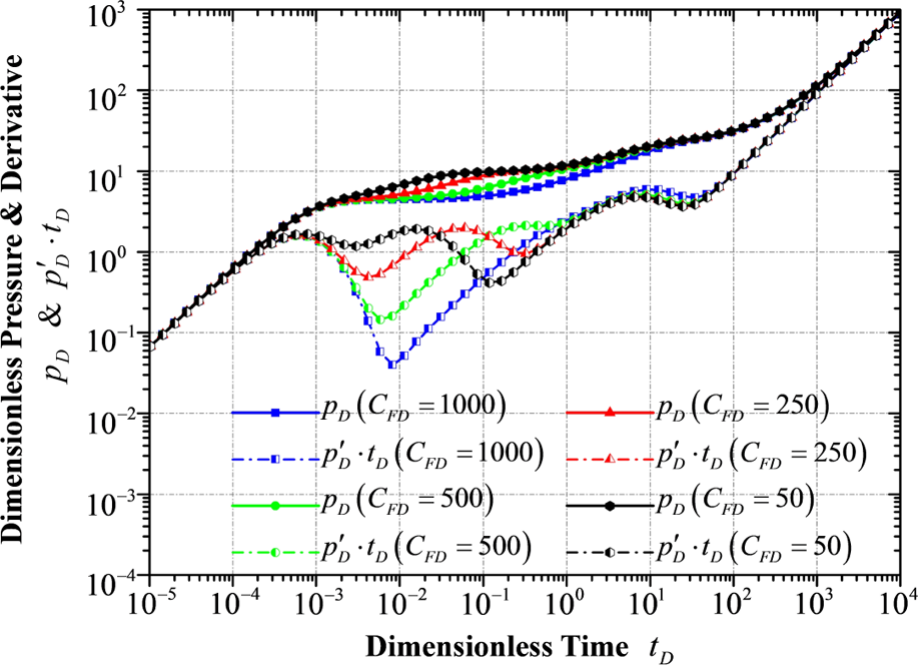
Transient pressure/pressure derivative response to different conductivities of hydraulic fracture.

It is pretty apparent that a larger hydraulic fracture conductivity will result in a smaller pressure derivative with respect to time at the early stage of the pressure derivative curve because a large hydraulic fracture conductivity is capable of offsetting or partially offsetting the wellbore storage influence (Figure 12). A larger hydraulic fracture conductivity indicates that more fluid will flow into the horizontal wellbore through the hydraulic fracture during the same unit of period, and it requires more fluid provided by the fracture system of the inner reservoir flow region, delaying the flow period in the fracture system or even affecting the interporosity flow from the matrix system to the fracture system in this flow region.

### 4.7 Response to Different Choking Skin Factors *S*_c_

**Figure 13:**
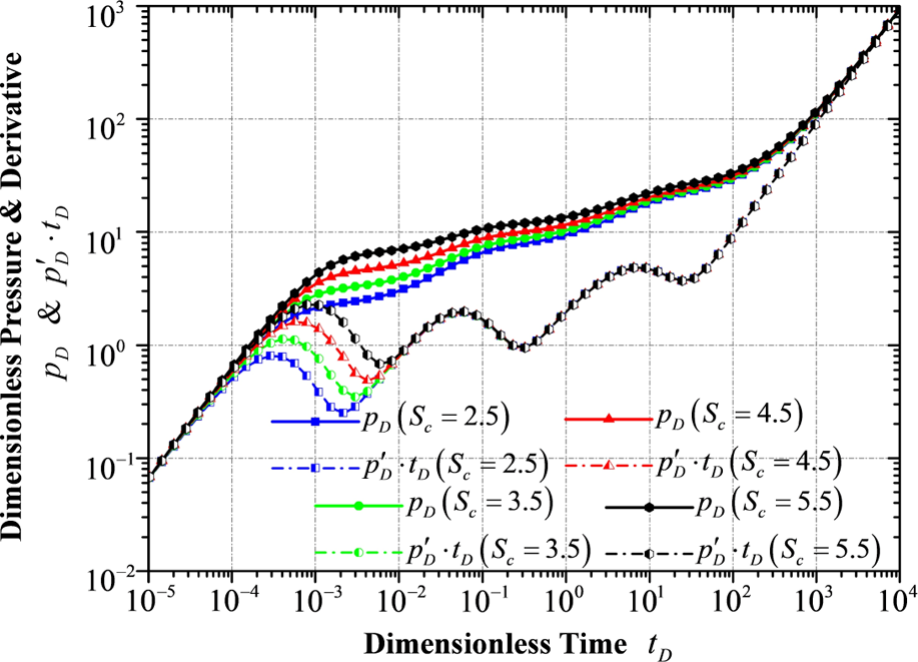
Transient pressure/pressure derivative response to different choking skin factors.

Figure 13 shows that the choking skin factor *S*_c_ mainly affects the wellbore storage stage and the transition flow period in the early-time pressure response. The choking skin factor typically refers to an additional pressure drop, consuming more reservoir energy as fluid flows into the horizontal wellbore in a convergent flowing pattern. In the well test interpretation, these two stages are mainly influenced by a combination of parameters, namely, *C*_D_·*e*^Sc^. Accordingly, the figure shows a larger dimensionless pressure as the choking skin factor increases.

### 4.8 Response to Different Wellbore Storages *C*_D_

**Figure 14:**
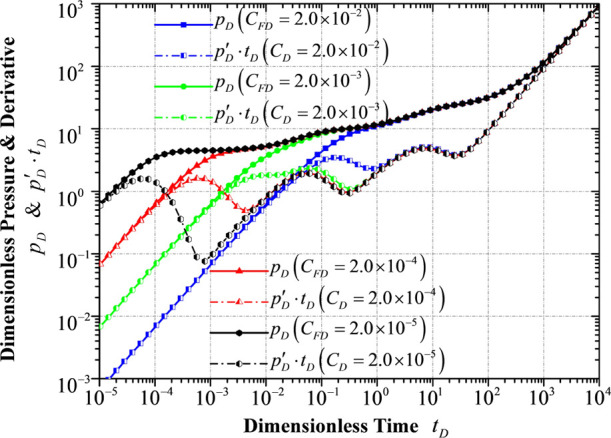
Transient pressure/pressure derivative response to different wellbore storage coefficients.

As illustrated in Figure 14, the wellbore storage effect typically influences the early and intermediate pressure response, showing that a larger wellbore storage coefficient leads to a longer elapsed time of wellbore storage effect on the whole production life and even delays the fluid flowing in the fracture system and the interporosity flow from the matrix system to the fracture system in the inner reservoir flow region.

### 4.9 Flow Regime Division

**Figure 15:**
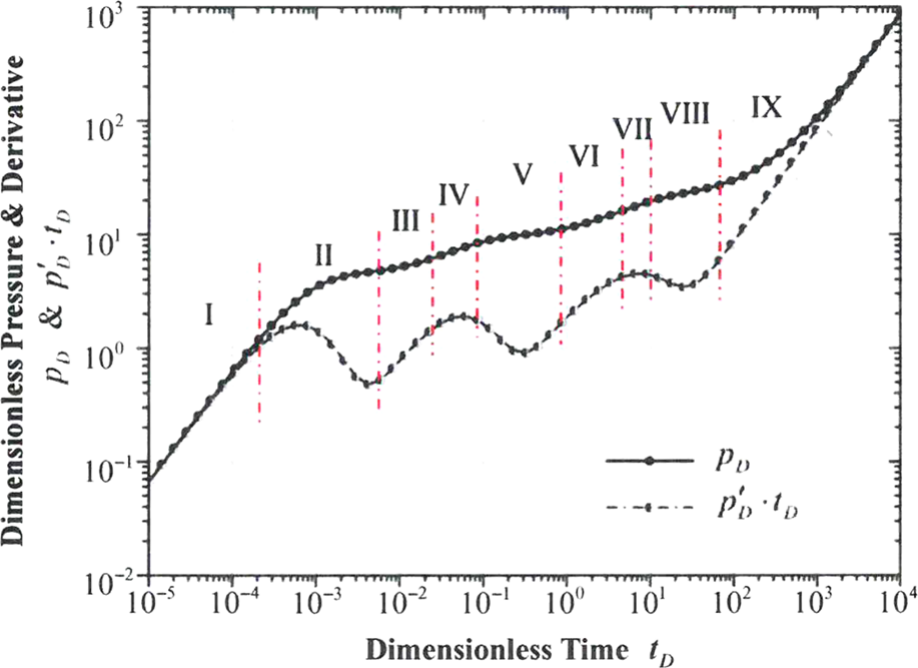
Nine different flow regimes of the TDPDP flow model.

Based on the model analysis and with the general data given in Table 4, Figure 15 shows the typical well test curves, which are generated and plotted using the MATLAB code. Based on the dimensionless pressure derivative curve, nine different flow regimes are observed, which are as follows.

**Table 4:** General Data for the TDPDP Flow Model

properties of the reservoir/fluid/horizontal well			
reservoir boundary, *x*_*e*_ (ft)	9000	outer reservoir fracture compressibility, *C*_*tO*_^*f*^, (psi^–1^)	3.0 × 10^–5^
inner reservoir size, *y*_*e*_ (ft)	800	wellbore radius, *r*_*w*_ (ft)	0.25
oil viscosity, μ, (cp)	5	length of horizontal wellbore, *L*_*w*_ (ft)	4500
initial HF permeability, *k*_*F*_^(*i*)^, (mD)	5.0 × 10^4^	inner reservoir matrix permeability, *k*_*I*_^*m*^, (mD)	3.0 × 10^–4^
HF permeability modulus, γ_*F*_, (psi^–1^)	1.5 × 10^–3^	inner reservoir matrix porosity, *ϕ*_*I*_^*m*^,	0.15
HF porosity, ϕ_*F*_, decimal	0.10	inner reservoir matrix compressibility, *C*_*tI*_^*m*^, (psi^–1^)	1.5 × 10^–5^
HF compressibility, *C*_*tF*_, (psi^–1^)	1.0 × 10^–4^	inner reservoir fracture permeability, *k*_*I*_^*f*^, (mD)	5.0 × 10^2^
HF height, *h* (ft)	250	inner reservoir fracture porosity, ϕ_*I*_^*f*^,	0.01
HF half-length, *x*_*F*_, (ft)	250	inner reservoir fracture compressibility, *C*_*tI*_^*f*^, (psi^–1^)	4.5 × 10^–5^
HF width, *w*_*F*_ (ft)	0.01	fractal dimension of the fractal-fracture network, *D*, dimensionless	2.05
outer reservoir matrix permeability, *k*_o_^*m*^, (mD)	2.0 × 10^–4^	Euclidean-embedding dimension, *d*, dimensionless	2.0
outer reservoir matrix porosity, ϕ_*o*_^*m*^,	0.20	connectivity index, θ, dimensionless	0.05
outer reservoir matrix compressibility, *C*_*tO*_^*m*^, (psi^–1^)	1.0 × 10^–5^	constant flow rate, *q*, STB/day	150
outer reservoir fracture permeability, *k*_*o*_^*f*^, (mD)	1.0 × 10^2^	wellbore storage coefficient, *C*, bbl/psi	2.0 × 10^–2^
outer reservoir fracture porosity, ϕ_*o*_^*f*^,	0.02	formation oil volume factor, *B*_*o*_, bbl/STB	1.25

**Regime I** is the stage of the wellbore storage effect. The pressure curve overlaps the pressure derivative curve. The unit slope of both curves is also observed in this figure. The elapsed time of this stage is mainly affected by the wellbore storage coefficient *C*_*D*_ and the fractal dimension of the fracture network *D*. Specifically, based on the model analysis in Section 2, a larger wellbore storage coefficient and a smaller fractal dimension will cause a longer elapsed time during this flow period.

**Regime II** is the transition flow period. The pressure derivative curve shows a “going-down” trend and the first hollow occurs on this curve. This stage is primarily influenced by the hydraulic fracture conductivity *C*_*FD*_, the fractal dimension of the fracture network, as well as the fractal connectivity index *θ* and the combination of parameters *C*_*D*_·*e*^2*S*_*c*_^.

**Regime III** is the flow period of the fracture system and the matrix system in the inner reservoir flow region, which is generally affected by a combination of parameters, including the wellbore storage coefficient *C*_*D*_, the choking skin factor S_c_, the hydraulic fracture conductivity *C*_*FD*_, the fractal connectivity index *θ*, and even a large hydraulic fracture permeability modulus γ_*FD*_.

**Regime IV** is the pseudo-radial flow period, a really short period before the interporosity flow from the matrix system to the fracture system in the inner reservoir flow region. In some typical scenarios, this stage may disappear.

**Regime V** is the interporosity flow stage in the inner reservoir flow region where the second hollow occurs on the curve of the dimensionless pressure derivative. This stage is generally influenced by the interporosity flow coefficient λ_*I*_, the storativity ratio ω_*I*_, even a large hydraulic fracture conductivity *C*_*FD*_, and a larger wellbore storage coefficient *C*_*D*_.

**Regime VI** is the flow period of the fracture system in the outer reservoir flow region, the unstimulated flow area. Extremely large hydraulic fracture conductivity *C*_*FD*_ and wellbore storage coefficient *C*_*D*_ may impact this flowing regime.

**Regime VII** is the pseudo-radial flow period before the interporosity flow from the matrix system to the fracture system in the outer reservoir flow region. In some typical scenarios, this stage may disappear.

**Regime VIII** is the interporosity flow stage in the outer reservoir flow region where the third hollow occurs on the curve of the dimensionless pressure derivative. This stage is primarily influenced by the interporosity flow coefficient λ_*O*_ and the storativity ratio ω_*O*_.

**Regime IX** represents a combination of the boundary effect and the effect of the hydraulic fracture permeability modulus, showing a sharp going-up trend in both the pressure curve and the pressure derivative curve. On account of the hydraulic fracture permeability modulus, the slope of the pressure derivative curve is larger than ONE (the boundary effect only generally stands for a unit slope in the curve of the dimensionless pressure derivative).

## 5 Conclusions

Based on the results obtained from this comprehensive study, the following conclusions are drawn.

(1) The trilinear dual-permeability and dual-porosity flow model is a novel field-applicable mathematical model for transversely fractured horizontal wells in naturally fractured oil reservoirs. This model comprises the inner reservoir flow region as a stimulated area where the dual-permeability model was introduced, and the outer reservoir flow region as a nonstimulated flow region where the integrated dual-porosity model was introduced.
(2) The newly developed mathematical model incorporates high heterogeneity caused by naturally developed highly disordered fractures, an innovative method of describing the non-Darcy flow effect to identify the anomalous diffusion of fluid through these fractures and an exponential law taking into account the high pressure-sensitivity influence on hydraulic fractures.
(3) The model also presents the sensitivity analysis for dimensionless bottom-hole flowing pressure and dimensionless pressure derivative response with respect to different key parameters, including the permeability modulus of hydraulic fracture, the fractal dimension, the fractal connectivity index, the storativity ratio, the interporosity flow coefficient, the hydraulic fracture conductivity, the choking skin factor as well as the wellbore storage coefficient. Furthermore, field data was applied, demonstrating a good consistency between theoretical parameters and well test interpretation data. Based on dimensionless pressure derivative curves, this model presents nine different flow regimes, which are very consistent with the findings from Jiang et al.^17^ and Zhao and Du,^18^ and will enhance well test interpretations in TDPDP reservoirs.
(4) This novel mathematical model can greatly enhance the classic trilinear flow model, which would play a theoretical guiding role in well test interpretation of the transversely fractured horizontal well in naturally fractured oil reservoirs.

## Appendix A: Mathematical Model for the Matrix System (Outer Reservoir)^50^

Following eq 11, the modified pseudo-interporosity flow rate *q*_o_^*m*→*f*^ from the matrix system to the fracture system in the outer reservoir flow region is typically defined as eq A1, which is concerned with the time-fractional derivative of the pressure difference between these two systems.

A1

The expression of the matrix shape factor in the outer reservoir flow region σ_*o*_^*m*^ is defined as eq A2.

A2

With respect to the definition of the dual-porosity model, the flow term ∇•(ρ*v*_o_^*m*^) is supposed to be neglected. As such, substituting eqs 12 and A1 into eq 1, it can be rewritten as follows.

A3

The total compressibility of the matrix system in the outer reservoir flow region *C*_*Ot*_^*m*^ is defined as eq A4.

A4

Using the dimensionless parameters, eq A3 will be rearranged into a concise form expressed as eq A5.

A5

In eq A5, the interporosity flow coefficient in the outer reservoir flow region λ_0_ is defined as eq A6.

A6

The combined parameter χ_*O*_^*m*^ is expressed as eq A7.

A7

where

A8

The storativity ratio in the outer reservoir flow region ω_*O*_ is defined as eq A9.

A9

The diffusivity coefficient η_*I*_ is expressed as eq A10 in the field unit.

A10

The Laplace transform, first introduced for problems of the flow through porous media by Van Everdingen and Hurst,^49^ is widely used for partial differential equations (PDEs). It aims to transform PDEs to ordinary differential equations (ODEs) in the broad sense, replacing the physical time, ***t***, by the Laplace time, or Laplace variable, ***s***. The transformed equation does make sense in the Laplace domain in the form of an ODE. Accordingly, eq A5 will be simplified as below using the Laplace transform.

A11

where

A12

## Appendix B: Mathematical Model for the Matrix System (Inner Reservoir)^50^

The modified pseudo-interporosity flow rate *q*_*I*_^*m*→*f*^ from the matrix system to the fracture system in the inner reservoir flow region is defined, which is also related to the time-fractional derivative of the pressure difference between these two systems as follows.

B1

Substituting eqs B1 and 23 into eq 22, it, as such, can be expressed as eq B2.

B2

where

B3

With the dimensionless definitions, eq B2 can be rewritten in the dimensionless form in the Laplace domain as follows.

B4

where the combined function *h*_*I*_^*m*^ is defined as eq B5.

B5

At the right-hand side of eq B5, the first combined parameter χ_*I*_^*m*^ is expressed as eq B6.

B6

The interporosity flow coefficient λ_*I*_ and the storativity ratio ω_*I*_ in the inner reservoir flow region can be defined as eqx B7 and B8, respectively.

B7

B8

The dimensionless parameter κ_*I*_^*m*^ refers to the permeability ratio of the matrix system in the inner reservoir flow region, which is expressed as eq B9.

B9

The outer and inner boundaries of the matrix system in the inner reservoir flow region can be mathematically expressed in the dimensionless form in the Laplace domain as follows.

**Outer Boundary Condition**

B10

**Inner Boundary Condition**

B11

The combination of eqs B4, B10, and B11 is the mathematical model of the matrix system in the inner reservoir flow region.

## Appendix C: Mathematical Model for the Fracture System (Inner Reservoir)^50^

The governing equation of the fracture system in the inner reservoir flow region can be expressed as follows.

C1

where

C2

With the dimensionless definitions, eq C1 would be rewritten as eq C3 in the Laplace domain.

C3

where the combined functions *g*_*I*_ and *h*_*I*_ are defined as eqs C4 and C5, respectively.

C4

C5

As assumed that the flow occurs in the *y*-direction in this region, the integration method, in this connection, is performed with respect to *x*_*D*_ from *x*_*D*_ = 0 to *x*_*D*_ = 1 (*x* = *x*_*F*_), and it yields

C6

where a nonflow interface (*x*_*D*_ = 0) is encountered, which is mathematically expressed as eq C7.

C7

As we assumed, all of the fluid flowing for the outer reservoir flow area would flow into the fracture system in the inner reservoir flow area. In this connection, at the interface between the outer reservoir flow region and inner reservoir flow region, the flow rate must be continuous and identical, which can be mathematically expressed by eq C8 in the dimensionless form as follows.

C8

Considering the outer region fracture system and eq C8, it yields

C9

Then, substituting eq C9 into eq C6, it yields

C10

where

C11

and

C12

The outer and inner boundaries of the fracture system in the inner reservoir flow region can be mathematically expressed in the dimensionless form as follows.

**Outer Boundary Condition**

C13

**Inner Boundary Condition**

C14

The combination of eqs C10, C13, and C14 is the mathematical model of the fracture system in the inner reservoir flow region.

## Appendix D: Mathematical Model for the Flow Region of Hydraulic Fracture^50^

A linearization method, mathematically expressed as eq D1, is introduced for eq 32

D1

Substituting eq D1 into eq 32, it yields

D2

According to the perturbation theory, ξ_*FD*_ can be expanded as eq D3.

D3

where γ_*FD*_^*n*^ξ_*FD*_^(*n*)^ is the *n*-order approximate solution to the perturbation theory.

On the basis of the Taylor expansion, the term  can be expanded as eq D4.

D4

where *O*(γ_*FD*_^*n*^ξ_*FD*_^*n*^) is the *n*-order round-off error in the specific expression of the Taylor expansion.

In terms of eq D2, we would select the zero-order approximate solution of eq D3 and the zero-order round-off error of eq D4 since γ_*FD*_ is relatively small based on the practical field test and measurement.

In this connection, eq D2 can be transformed into eq D5 in the Laplace domain.

D5

Following a similar pattern of performing integration with respect to *y*_*D*_ from *y*_*D*_ = 0 to , it yields

D6

where

D7

At the interface between the inner reservoir flow region and the region of hydraulic fracture, the flow rate must be continuous and identical, and the mathematical formula can be written in the dimensionless form as follows.

D8

After rearranging eq D8, it yields

D9

D10

where

D11

Based on the Taylor expansion, the following equation can be expanded.

D12

In this connection, eq D10 can be rewritten as eq D13 as we select the zero-order round-off error of eq D12.

D13

Substituting eq D13 into eq D6, it yields

D14

where

D15

The general solution to eq D15 is expressed as follows.

D16
